# Assessment of three large-scale depopulation methods for swine

**DOI:** 10.1371/journal.pone.0320217

**Published:** 2025-03-25

**Authors:** Janice Y. Park, Magnus R. Campler, Ting-Yu Cheng, Brad L. Youngblood, Dawn Torrisi, Michael D. Cressman, Justin D. Kieffer, Todd E. Williams, Andréia G. Arruda, Gary A. Flory, Daniel P. Hougentogler, Jeff Hill, Lucia Hunt, Albert Canturri, Marie R. Culhane, Jesse Miller, Andrew S. Bowman

**Affiliations:** 1 Department of Veterinary Preventive Medicine, The College of Veterinary Medicine, The Ohio State University, Columbus, Ohio, United States of America; 2 Office of the Attending Veterinarian, The Enterprise for Research, Innovation and Knowledge, The Ohio State University, Columbus, Ohio, United States of America; 3 Department of Animal Sciences, The College of Food, Agricultural, and Environmental Sciences, The Ohio State University, Columbus, Ohio, United States of America; 4 Pipestone Research, Pipestone M.N., United States of America; 5 Agricutural Emergency Service Incorporated, Newark, D.E., United States of America; 6 Office of Emergency Preparedness & Response, Minnesota Department of Agriculture, Saint Paul, Minnesota, United States of America; 7 College of Veterinary Medicine, University of Minnesota, Saint Paul, Minnesota, United States of America; 8 University of Minnesota School of Statistics, Minneapolis, Minnesota United States of America; Universidade do Porto Instituto de Biologia Molecular e Celular, PORTUGAL

## Abstract

The threat of foreign animal disease outbreaks to U.S. swine herds warrants effective and readily available depopulation methods. Current American Veterinary Medical Association-recommendations using preferred physical methods for swine depopulation are unsuitable for large commercial swine herds. Our objectives were to assess and compare the efficacy and performance of three suggested large-scale depopulation methods: 1) medium-expansion water-based foam, 2) prototype high-expansion nitrogen foam and, 3) carbon dioxide gas for finisher pigs under field conditions. Out of 793 finisher pigs included in the study, 84 were implanted with bio-loggers recording electrocardiogram and pig movement data. Aversive pig behaviors were collected manually on a group level for each depopulation method. A subsample of pigs from each method were examined post-mortem for lesions and compared to a reference group of nine pigs euthanized with pentobarbital. Depopulation method assessments included container fill time, the number of aversive pig behaviors observed during depopulation, overall pig movement intensity, time to cessation of movement, time to and cause of cardiac arrest, and respiratory lesions. No difference in fill times between water-based foam and nitrogen foam was observed. The total number of aversive swine behaviors was higher for carbon-dioxide compared to both foam methodologies (*P* <  0.01). The total pig activity was higher in water-based foam compared to nitrogen foam (*P* =  0.02) and carbon-dioxide methods (*P* =  0.01). The mean time to cessation of movement was significantly shorter for water-based foam and nitrogen foam compared to carbon-dioxide (*P < * 0.01). No differences in cardiac activity were observed. Water-based foam pigs had increased odds of distal trachea occlusions compared to other methods. All depopulation methods demonstrated high efficacy with a 100% mortality rate. The results from this study support large-scale water-based foam, nitrogen foam and carbon dioxide as viable AVMA depopulation guideline candidates for swine.

## Introduction

Depopulation is defined by the American Veterinary Medical Association (AVMA) as “the rapid destruction of a population of animals in response to urgent circumstances with as much consideration given to the welfare of the animals practicable” [1]. The emphasis on ‘rapid destruction’ is especially important for the swine industry, which is under the constant threat of foreign animal disease (FAD) outbreaks but where options for rapid animal destruction for disease containment are limited. Some of the preferred physical methods currently recommended in the AVMA’s guidelines for depopulation of animals (e.g., penetrating captive bolt or gunshot) are prone to low efficiency, operator fatigue/trauma, or high risks of biohazardous contamination [1,2]. On the other hand, certain methods, such as the AVMA’s “permitted under constrained circumstances*”* ventilation shutdown plus (VSD+), have been modified to accommodate depopulating larger numbers of swine. However, VSD + risks prolonged periods of discomfort until death, raising concerns regarding animal welfare [3]. The current size and scale of modern swine production limits the US swine industry’s ability to efficiently depopulate large populations of swine with the current approved methods at hand. Therefore, it is necessary to develop additional swine depopulation methods that are effective and reliable for rapidly depopulating large swine herds within the US industry.

The use of inert gases for pig stunning is currently permitted in the European Union [4], and carbon dioxide (CO_2_) is widely used within the swine industry globally during slaughter and small batch, on-farm euthanasia. As such, CO_2_ gas is a method for swine depopulation that can operate in field conditions using a system involving an animal-holding chamber filled with gas [5]. Exposure to CO_2_ induces intracellular acidosis, which disrupts neural activity and results in unconsciousness and subsequent death [6]. The use of CO_2_ may facilitate large scale depopulation with the advantage of minimal handling of animals and rapid loss of consciousness [7] but also comes with concerns regarding pig aversion during exposure [2,8,9]. Carbon dioxide gas reliably produces unconsciousness and death in swine at 80% to 90% concentrations when used in commercial dip-lift stunning systems, with higher concentrations leading to faster time to unconsciousness [10,11]. To reduce the amount of stress on the pigs during the depopulation procedure, the AVMA guidelines for depopulation of animals recommend a minimum CO_2_ displacement rate of 20% of the container volume per minute for five min [1]. This recommendation is based on findings reporting unconsciousness and death within two and ten min after CO_2_ exposure using animal-holding chambers, respectively [12]. However, CO_2_ depopulation may encounter issues with sourced gas availability and the safety of personnel and environmental safety [13].

In 2006, water-based foam (WBF) was approved for poultry depopulation by the United States Department of Agriculture (USDA) and the AVMA [14]. The emergency depopulation of floor-reared poultry flocks using water-based foam has been shown to be an effective method by means of inducing occlusion hypoxia, leading to unconsciousness and subsequent death [14]. Despite the wide acceptance of WBF for poultry depopulation, water-based or gas-infused foams are not currently included in the AVMA guidelines for depopulation of animals for other agricultural species, such as swine. Recent studies focused on swine have reported high efficacy with rapid loss of consciousness and death using WBF in sows [15], suckling [16], and finisher pigs [16–18]. Additionally, testing and refinement of WBF protocols have recommended a minimum of 7.5-minute dwell time and a minimum foam fill height of twice the height of the dorsal apex of the pig’s back when standing horizontally [18].

Research on the addition of inert gases such as helium, argon or nitrogen to high-expansion foams has shown great potential, as gas filled bubbles increase the displacement rate of oxygen to create anoxic conditions [19,20]. Foam bubbles containing inert gas break apart, releasing the gaseous elements or compounds, and rapid death occurs by achieving acute cellular anoxia in the brain [21]. For instance, nitrogen-infused foam (N_2_F) has been successfully implemented for poultry [22], suckling- [23], weaner- [24], and finisher pigs [25]. In addition, the use of nitrogen gas (N_2_), has been promoted as a more welfare friendly alternative to CO_2_ due to its less adverse properties when inhaled [26]. The task of exploring and assessing additional large scale depopulation methods is paramount as a part of comprehensive contingency planning for emergency situations, such as FAD outbreaks that threaten swine populations. An increased arsenal of depopulation methods improves local and regional opportunities for disease control. During outbreaks involving multiple premises, competition of supplies and specialized equipment will be high, making the availability of various options critical. Thus, the objectives of this study were to evaluate three depopulation methods (WBF, N_2_F and CO_2_) to investigate 1) their efficacy and performance; 2) any method difference in activity levels, behavior, time to cessation of movement and cardiac activity; and 3) compare post-mortem lesions and accumulation of froth within the respiratory tract as evidence of death by occlusion hypoxia between swine depopulated during field conditions and swine euthanized by pentobarbital (PB).

## Materials and Methods

### Study approval and animal subjects

The WBF part of this study was approved by the Institutional Animal Care and Use Committee (IACUC) at The Ohio State University, animal use protocol 2020A00000036-R1. The N_2_F and CO_2_ parts of this study were approved under IACUC animal use protocol 2023-8 for Pipestone Research. Finisher pigs (N =  793) of mixed sex and weight (range: 90-135 kg) were included in this study. All pigs were housed and maintained by the producer prior to use in the study and were divided into groups for each method (WBF, N_2_F, or CO_2_) based on convenience as the barn was emptied from one side to the other during the depopulation event. Each method consisted of four replicates. An additional group of nine conveniently selected pigs was created for individual euthanasia by lethal injection to serve as post-mortem lesion controls. This PB group was euthanized with Euthasol^®^ (390 mg pentobarbital and 50 mg phenytoin sodium mixture/ ml) using 1.0 ml/4.5 kg body weight. Method-specific replicates were completed in succession and ranged from 59 to 75 pigs (Table 1). After each replicate completion, the trailer was repositioned to a designated offloading area on site. All pigs were examined by veterinarians for any signs regaining consciousness and confirmation of death. Carcasses were disposed of on-site through composting.

**Table 1:** Time (mm:ss) to reach designated fill levels, outlined by replicates of pigs depopulated using water-based foam (WBF), nitrogen-foam (N_2_F), and carbon dioxide (CO_2_).

Method	Replicate	Level of Shoulder (mm:ss)	Level of Head (mm:ss)	Complete Fill (mm:ss)
**WBF**	1	00:13	00:20	00:56
	2	00:07	00:21	01:01
	3	00:13	00:23	01:07
	4	00:18	00:26	01:01
**N**_**2**_ **F**	1	00:24	00:38	01:32
	2	00:10	00:15	01:04
	3	00:09	00:31	01:42
	4	00:33	00:39	01:14
**CO**_**2**_	1	N/A	N/A	06:08
	2	N/A	N/A	05:00
	3	N/A	N/A	05:00
	4	N/A	N/A	05:00

### Bio-logger implantation, and movement data collection

For each replicate, seven pigs were implanted with bio-loggers (DST-Centi-HRT ACT, Star-Oddi, Garðabær, Iceland) programmed to obtain continuous electrocardiogram (ECG) and movement data at 15 s intervals. Bio-loggers were surgically implanted within the axillary subcutaneous space using methods described by Lorbach et al. (2021)[15]. After each replicate, bio-loggers were removed from carcasses for data retrieval.

### Water-based foam generation and application

All pigs were loaded from the swine barn into a 64.5 m^3^ (dimensions 12.2 ×  2.36 ×  2.24 m, length, width, and height) hydraulic rear-loading modified rendering trailer. The trailer was filled from above by pumping 1% solution of PHOS-CHECK WD881 Class A foam concentrate (Perimeter Solutions, Rancho Cucamonga, CA, USA) through three medium-expansion dual-expansion handline foam nozzles (KR-M4, ANSUL, Marinette, WI, USA). Each nozzle was fed by its own high-pressure water pump. The full details for the foam generation setup and trailer specifications are detailed in Arruda et al. (2022) [17]. All pigs remained undisturbed in the foam for a dwell time of 7.5 min.

### High expansion nitrogen-based foam

All pigs were loaded from the swine barn into the same 64.5 m^3^ modified rendering trailer used for the WBF depopulation trial. The trailer was filled with high-expansion N_2_F with an expansion ratio of foam to water of > 250:1 consisting of nitrogen-filled bubbles approximately 15 mm in diameter delivered from a 50 cm diameter prototype N_2_F generator (prototype designed by Agricultural Emergency Services Inc (AES Inc, Newark, DE, USA), which included dual nucleation sites for optimal bubble expansion and sizing. The N_2_F generator was connected to a pump trailer and a commercial nitrogen vaporizer truck. The AES Inc pump trailer supplied water, and a 2% foam concentrate solution of PHOS-CHECK WD881 Class A foam concentrate (Perimeter Solutions, Rancho Cucamonga, CA, USA) for the N_2_F generating systems using a Darley water pump (model 2 1/2 AGE 48K, Darley, Itasca, IL). The pump was used to supply the required pressure and flow and driven by a 35.8-kW (48hp) Kubota diesel engine (Kubota, Lincolnshire, Il, USA). A 2% solution of water-foam concentrate is pumped to the generator where the N_2_ and water-foam concentrate solution are combined. The foam concentrate was proportioned into the water source prior to the pump inlet and allowed to mix throughout the system. Foam concentrate was provided to the water system by a Dostec-40 (ITC Dosing Pumps, Barcelona, Spain) static flow dosing pump. The water/ foam solution was delivered to the N_2_F generators via 33.3 meters (100 feet) of 5 cm (2”) commercially available fire hose. The N_2_ was delivered separately to the N_2_F generators from a commercial service provider operating a 254,000 l/min (540k scfh) diesel powered pump truck. A 2.54 cm diameter and 33.3 m long high-pressure nitrogen hose was used to deliver the high flow, low pressure gas to the generator input. After successful filling of the container a dwell time of 7.5 min was used before trailer movement was allowed.

### Carbon dioxide inhalation

Pigs were loaded from the swine barn through a rear lift gate into a standard frameless end-dump trailer. The trailer was modified by installing aluminum hinged ceiling panels to minimize the volume of gas required per fill. This ceiling provided an interior volume of 29.3 m^3^ (dimensions 12.2 ×  2.4 ×  1.0 m, length, width, and height). A commercial tanker of liquid CO_2_ was connected to two serially connected non-electric ambient air (capable of moving 425 m^3^/per hour) vaporizers. A pressure regulator was installed upstream of the vaporizers to reduce the inlet pressures. A second pressure regulator was installed at the outlet of the vaporizer to maintain proper outlet pressure. Gaseous CO_2_ then flowed through a four-hose manifold and into four inlet ports at floor level of the trailer. Four outlet vents at ceiling height were also confirmed open at this time. The trailer was filled from the bottom by passive gas expansion. An oxygen sensor was attached to monitoring ports on the trailer and evaluated to ensure levels inside the trailer were below 10% O_2_ after a 5-minute fill time using 2000-3000 gallons of liquid CO_2_ per fill, corresponding to a CO_2_ inflow rate equivalent to 24% of the container volume per minute. Once CO_2_ fill standards were achieved, the supply of CO_2_ gas was stopped, and the inlet ports and outlet vents were both closed. Specifications for CO_2_ trailer construction and use are detailed in Pepin et al. (2022)[27]. All pigs remained undisturbed in the trailer for a dwell time of 10 min.

### Individual euthanasia with lethal intravenous injection of pentobarbital

Nine pigs were conveniently selected to serve as post-mortem lesion controls. Each of them was manually restrained and individual euthanasia was performed by an experienced licensed veterinarian (T.E.W; Pipestone) who intravenously injected into the cranial vena cava a lethal dose (1 ml/ 4.5 kg) of a pentobarbital-combination product (pentobarbital sodium 300 mg and phenytoin sodium 50 mg) with an 18-gauge needle and 30 ml syringe. After the pig was restrained via snaring, the needle was inserted at a 45^o^ angle into the vena cava approximately 1“ cranial to the sternum a little lateral and to the right of the midline. Blood flow was stopped by applying finger pressure on the blood sampling site for approximately 30 seconds.

### Behavioral data collection

An animal welfare specialist (M.R.C; The Ohio State University) served as a designated observer to monitor the behaviors of pigs during each trial. For the WBF and N_2_F trials, the observer was positioned on top of the trailers’ cantilevered viewing platform allowing for a full top-down view of the pigs. Behaviors monitored were vocalization (audible vocalizing), escape attempts (climbing or jumping at the side of trailer or another pig), resurfacing (any visible part of the body breaking the foam surface during or after fill completion) and the time to the last visible or audible animal movement post-agent initiation for each method.

Behavioral observations were manually tallied using a pen and paper based on the number of occurrences on a group level during a 30 s time frame from the start of agent initiation until the foam level covered the pigs to a depth where animals were no longer visible through the foam. Any auditory cues such as movements within the trailer were recorded until the end of the dwell period (during which the trailer and pigs were left undisturbed).

For the CO_2_ trial, the observer was positioned on the ground next to the trailer wall. The observer was limited to auditory cues based on the study setup being covered and animals being out of view. Therefore, only the number of distinct vocalizations and the time to the last audible movement within the CO_2_ trailer vocalizations between the time of agent initiation to the end of dwell-time was recorded.

### Individual identification, body weight collection and post-mortem examinations

Ante-mortem bodyweights were collected from 68 pigs (CO_2_, N =  25; WBF, N =  25; N_2_F, N =  9; and PB =  9). Due to time constraints and field logistics, only 9 out of the anticipated 25 N_2_F pigs were included for post-mortem examination. All pigs were weighed inside the barn before being loaded for depopulation by walking onto portable scale with digital display (WayPig^®^ Digital Market Hog Scale, Raytec LLC, Ephrata, PA. The scale was calibrated between groups with a standard 20 kg weight. After confirmation of death by the assigned method, the individually identified pigs were carried to the necropsy area where the pigs were placed onto the same portable scale to obtain the post-mortem weight. All pigs were weighed and examined for lesions post-mortem. The post-mortem exam was performed by a board-certified veterinary pathologist (A.C., University of Minnesota) with the assistance of a veterinary diagnostician (M.R.C, University of Minnesota) and veterinary technician (J.Y.P; The Ohio State University). A subset of pigs from each method group was individually identified with a numbered ear tag, weighed before death, and examined for post-mortem lesions by veterinary pathologists and a diagnostician (A.C., M.R.C; University of Minnesota).

### Data management and statistical analysis

#### Time to cessation of movement.

As a conservative proxy measurement of time to unconsciousness in pigs, we used the time to cessation of movement after the start of agent initiation to determine movement-derived external acceleration (EA) data from bio-loggers. The obtained EA measurements corresponded to pig movement as tri-axial accelerations, or intensity of movement in any spatial direction, which were analyzed according to methods previously outlined by Arruda et al. [17]. Baseline EA readings were normalized to the standard acceleration of gravity (9.8 m/s^2^). The EA threshold for cessation of pig movement was estimated using EA values between the initiation of the depopulation agent and the end of the 7.5-minute dwell time and calculated as the sum of the third quantile and 1.5 times the interquartile range, as established in prior studies [16,17]. The threshold was calculated for each implanted pig to determine “outlying” EA values, i.e., instances of plausible pig movement controlling for background noise such as wind effects on the trailer or false movements caused by activity by adjacent and conscious pigs. That is, any EA value above the individually calculated threshold (outlier) for each pig was considered indicative of pig movement. Times to cessation of movement (COM) were defined as the difference between the first successive timepoint after the first EA value below the assigned threshold—with no successive values over threshold for a five-minute period—and the start of depopulation agent application as described in Kieffer et al. [16]. Descriptive statistics, including mean, median, first and third quartile, interquartile range, standard deviation (SD), were calculated and presented in text. A Kaplan-Meier survival curve was created to depict times to COM events per depopulation method. A mixed negative binomial regression model with Bonferroni corrections to adjust for multiple comparisons was used to investigate the effect of depopulation method (CO_2_, WBF and N_2_F) on time to COM. Depopulation method was used as a fixed effect, with replicate as a random effect.

#### Movement intensity.

**Fig 1:**
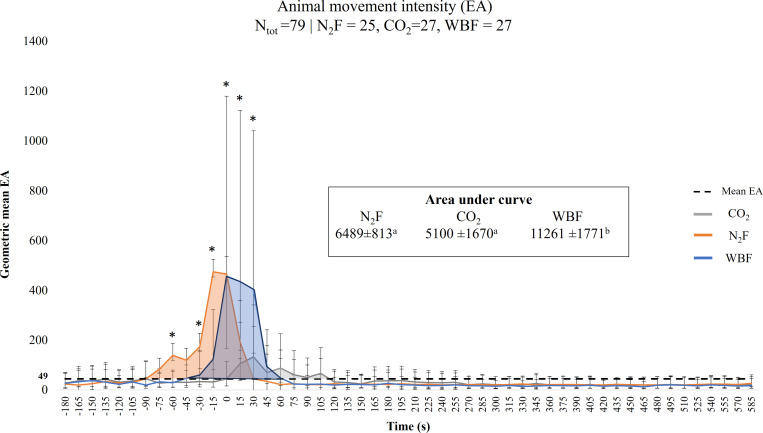
Geometric mean [95% CI, lower, upper] external animal movement intensity (EA) for finisher pigs (N =  79) for nitrogen foam (N_2_F), carbon dioxide gas (CO_2_), and water-based foam (WBF). Depopulation agent application occurred at 0 s. A CO_2_ inflow rate equivalent to 24% of the container volume per minute was utilized to achieve the complete fill time. Geometric mean (±SD) for the area under curve (AUC) for each method is calculated from the first EA peak above until the last peak above the geometric mean threshold (EA_threshold_ =  49). Differing AUC superscripts (^a,b^) indicate a significant difference *P* <  0.05. Differences in activity levels between methods for individual time points are indicated as (*) for *P* <  0.05.

The EA from 180 s (3 min) prior to the initiation of agent initiation to 585 s (+10 min) post-agent initiation was analyzed at 15 s intervals using a linear mixed model with repeated measures for each method across each time point with pig nested within replicate as random effect. The EA data was transformed using a log10 transformation to normalize residuals. Model derived back transformed geometric means and 95% confidence intervals were used for EA visualization (Fig 1). Post-hoc pairwise comparisons were performed to estimate the difference in EA between methods and Bonferroni correction was implemented to adjust for multiple tests. Total movement intensity throughout the depopulation process was calculated for each method across all four replicates, using area under the curve (AUC) between the first EA peak above to the last EA peak above a calculated geometric mean EA threshold (averaged across all method) (dashed line, Fig 1). A one-way analysis of variance (ANOVA) was used to detect differences in mean total AUC between methods.

#### Electrocardiogram analysis.

The time to and the type of cardiac arrest was determined by an experienced experimental surgery and clinical veterinarian (B.L.Y; The Ohio State University) using the ECG readings derived from the bio-loggers. Tracings were reviewed using Mercury software program v.6.30 (Star Oddi, Garðabær, Iceland). Baseline patterns were established by reviewing ECG readings taken prior to depopulation agent initiation. The time until fatal rhythm was determined by the difference between the onset of foam initiation and the beginning of a persistent (>3 min) fatal rhythm. The type of cardiac arrest was determined by the presence of a fatal arrhythmia that has been documented findings in swine models of asphyxial cardiac arrest [28].

Statistical analyses for movement, behavior, and ECG data were conducted using Stata v17.0 (StataCorp, College Station, TX, USA).

#### Post-mortem lesion assessment and data analysis.

For each pig, time of death, and time of post-mortem examination was recorded to calculate time lapsed (min) between confirmed death and necropsy. We investigated whether the amount of froth (thick pink liquid containing bubbles derived from pulmonary edema) within the tracheal lumen, a manifestation of pulmonary edema was consistent with occlusion hypoxia. The froth amount was subjectively scored 0, 1, 2, 3, 4 with 0 indicating no froth (dry tracheal lumen), 1 =  froth present but not occluding the lumen, 2 =  froth present but partially occluding the lumen and tracheal bifurcation still visible, 3 =  froth present, occluding the lumen, and to a level < 1 cm above the bifurcation, and 4 =  froth present, lumen fully occluded, and to a level > 1 cm above the bifurcation. The right cranial lung lobe was dissected and a section of the lung, a thickness of 1 cm, was fixed in 10% neutral buffered formalin for histopathology examination by board-certified veterinary pathologist (A.C., University of Minnesota), who was blinded to the method groups. Differences in the presence of froth in the trachea, i.e., froth scores and histopathologic lung lesions were compared between method groups using R Statistical Software (v4.3.1; R Core Team 2023). Exploratory analyses were performed initially for discovery purposes and, where indicated in the results below, proportional odds models and Dunnett’s tests for significance were performed.

Histological lung lesions previously described [29,30] as characteristic of asphyxiation, namely alternating collapse with emphysema/overinflation, alveolar hemorrhage, alveolar edema, alveolar septa congestion, interstitial (broncho-vascular) edema with erythrocyte diapedesis, bronchiolar dilation, interlobular septa edema, interlobular septa hemorrhage, and interlobular septa dilation were subjectively assigned lesion severity scores of (0) no lesions, (1) mild, (2) moderate, and (3) marked. In addition, the presence of other lung lesions suggestive of concurrent porcine respiratory or systemic diseases, specifically interstitial pneumonia, suppurative bronchopneumonia, bronchointerstitial pneumonia, and bronchus-associated lymphoid tissue hyperplasia, were recorded but not scored. Therefore, to determine if there were differences in the presence and severity (score) for alveolar hemorrhage or any of the lesions of interest were dependent on a concomitant pneumonia, an exploratory Chi-Squared test was performed.

Since our study had small sample sizes and variation in the sample sizes between groups, the *P*-values obtained from the Chi-Squared test were interpreted as suggestive only and needed further analysis. Any lesion covariate with a *P* <  0.10 was considered to be dependent on concomitant pneumonia being also present in the lung. Therefore, alveolar hemorrhage, interstitial edema, congested alveolar septa, foam, and emphysema were identified as lesions associated with asphyxiation and not pneumonia. Finally, Spearman’s ρ (rho) correlation coefficients were calculated to explore any positive or negative relationships between the ordinal lung lesion scores with other lung lesions (Supplemental Table 1). Statistical significance was declared at *P* <  0.05 and any tendencies as 0.05 ≤  *P* <  0.1.

## Results

### Method-specific logistics

All methods resulted in 100% mortality of their respective groups after a 7.5-minute dwell time. Mean (±SD) total container fill times (mm:ss) were 01:01 (±00:05) for WBF, 01:23 (±00:17) for N_2_F, ands 05:17 (±00:34) for CO_2_ (Table 1). Mean (±SD) time to fill to the level of the animals’ shoulder was 00:13 (±00:05) for WBF and 00:19 (±00:12) for N_2_F (Table 1). Mean (±SD) time to fill to the level of animals’ head was 00:23 (±00:03) for WBF, and 00:31 (±00:11) for N_2_F.

### Time to cessation of movement

**Fig 2:**
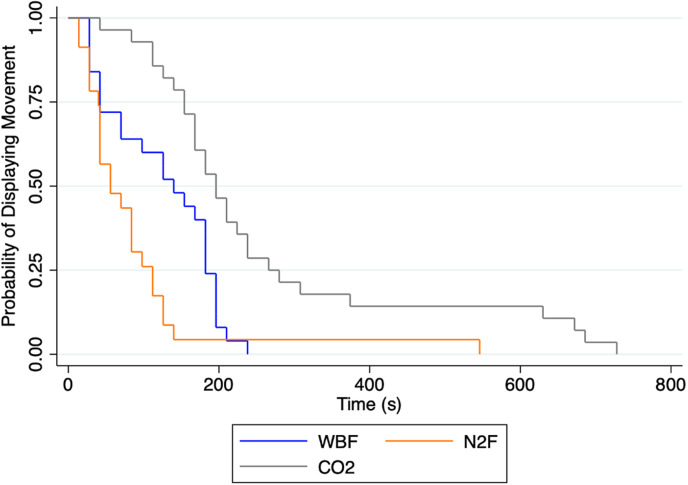
Kaplan-Meier survival curve showing pigs’ (N =  **79) probability of consciousness, as a measurement of displaying movement, by three depopulation methods 1) water-based foam (WBF, N** =**  27), 2) nitrogen foam (N**_2_**F, N** =**  25), and 3) carbon-dioxide gas (CO**_2,_ **N =  27).**

Results from the mixed negative binomial regression model, accounting for replicate as a random effect, revealed that the (mean [95% CI]) time to COM was shorter in N_2_F (1:23 [0:58-2:01] and WBF 2:05 [1:27-2:59] compared to CO_2_ (4:05 [2:53-5:47] (*P* <  0.01), while no difference was observed between N_2_F and WBF (*P* =  0.10). As visualized in the Kaplan-Meier survival curve, animals depopulated with CO_2_ had the longest time to COM followed by WBF, with N_2_F having the shortest time to COM (Fig 2). Descriptives for COM for each method and replicate are presented in Table 2.

**Table 2:** Descriptive statistics for time (mm:ss ±  SD) to cessation of movement (COM) and external activity (EA, milli-gravity (m*g*) [*g* = acceleration of gravity or 9.8 m s^ − 2^) for quartiles Q1 and Q3 as well as the interquartile range (IQR [Q3-Q1for finisher pigs (N =  79) depopulated using water-based foam (WBF), nitrogen-foam (N_2_F), and carbon dioxide (CO_2_).

Method	Replicate	N	Time to COM (m:ss ± SD)	Median EA (m*g*)	EA Q1 (m*g*)	EA Q3 (m*g*)	EA IQR (m*g*)
**WBF**	1	6	1:35 ± 1:12	26.0	11.5	46.0	34.5
	2	7	1:44 ± 1:14	21.0	9.0	35.0	26.0
	3	6	2:41 ± 0:53	29.0	16.8	43.0	26.2
	4	6	2:27 ± 1:15	41.0	23.0	68.0	45.0
**N**_**2**_ **F**	1	7	1:10 ± 0:38	35.0	16.0	47.0	31.0
	2	7	0:51 ± 0:31	32.0	15.0	55.8	40.8
	3	6	2:34 ± 3:41	34.0	10.0	43.0	33.0
	4	6	1:26 ± 0:49	19.5	11.0	31.2	20.2
**CO**_**2**_	1	7	4:02 ± 0:46	24.0	7.0	47.0	40.0
	2	7	3:19 ± 1:23	24.0	15.0	41.0	26.0
	3	7	7:18 ± 5:03	34.0	22.0	54.0	32.0
	4	7	2:36 ± 1:02	33.5	19.0	54.0	35.0

### Intensity of movement

Based on mean EA obtained from bio-loggers to approximate animal movement, each method displayed distinct movement patterns. The majority of N_2_F pig movement occurred in the period prior to the start of N_2_F initiation, with surges preceding the timepoint of agent application (Fig 2). Water-based foam pigs displayed their majority of intense movement between 30 s prior to 45 s after foam initiation (Fig 2). The CO_2_ pigs displayed an overall lower EA intensity but through a prolonged period predominantly observed post-gas initiation. A higher total geometric mean (±SE) AUC was observed for WBF compared to N_2_F (11261 ± 1771 vs. 6489 ±  813, *P* =  0.02) and WBF and CO_2_ (11261 ±  1771 vs. 5100 ±  1670, *P* =  0.01) but no difference between or N_2_F and CO_2_ was observed (6489 ±  813 vs. 5100 ±  1670, *P* =  0.46) (Fig 2). The highest observed geometric mean EA [95% CI, lower, upper] for each method was at time point -15 s for N_2_F (474 [28, 71]) and 0 s for WBF (458, [290, 722]) and 30 for CO_2_ (132 [84, 209]), (Fig 2).

### Cardiac activity

Data was sufficient for review in all implanted animals, except for one pig in the N_2_F group. The times to fatal arrhythmias tended to be longer for WBF compared to CO_2_ (9.6 ±  2.3 vs. 8.9 ±  3.0 min, *P* =  0.38). No differences in time until fatal arrhythmias were observed between WBF and N_2_F (9.6 ±  2.3 vs. 9.0 ±  2.8, *P* =  0.40) or N_2_F and CO_2_ (9.0 ±  2.8 vs. 8.9 ±  3.0 min, *P* =  0.96). Fatal arrhythmias identified included asystole, ventricular fibrillation, third degree atrio-ventricular block, and pulseless electrical activity (electro-mechanical dissociation). WBF and N_2_F animals predominantly developed ventricular fibrillation (70.4% and 76.9%, respectively) while CO_2_ predominantly developed asystole (75%). A single animal in the CO_2_ group had pulseless electrical activity 15 min post-foam initiation when confirmed deceased.

### Behavioral data

Due to the design of both WBF depopulation methods, behavioral observations were limited to the time span between foam initiation until pigs were covered in foam. In addition, the CO_2_ method used a covered and sealed trailer to maintain the CO_2_ concentration, thus preventing any visual observation of pigs and vocalizations. Vocalizations could not be assessed for N_2_F due to the degree of background noise from nitrogen foam generators. Therefore, the only assessment available for comparison across all three methods was the time from agent initialization until the last heard movement post agent initialization. The CO_2_ group had a higher number of recorded vocalizations (N =  173) compared to WBF (N =  18). Mean (±SD) elapsed times until the first observed response from animals after agent initialization were 00:05 ( ± 00:03), 00:05 ( ± 00:10), and 00:03 ( ± 00:05) for WBF, N_2_F, and CO_2_, respectively. For the visual observations, N_2_F-pigs displayed 22 resurfacings and >  30 escape attempts across the four replicates compared to one resurfacing event and nine escape attempts for WBF pigs. For the last movement post-agent initialization (mean ±  SD), N_2_F-pigs ceased to move the soonest (82 ±  2.2 s), followed by WBF (155 ±  46.6 s) and CO_2_ (287.5 ±  3.0 s). Behavioral data for each method and replicate can be found in Table 3.

**Table 3:** Descriptives of total counts (n) of aversive behaviors (resurfacings, escape attempts, vocalizations) and the time (s) to the last audible movement (LAT) post method agent initialization, by replicate per depopulation method for water-based foam (WBF), nitrogen foam (N_2_F) and carbon dioxide (CO_2_).

Method	Replicate	Pigs	Resurfacings	Escape attempts	Vocalizations	LAT
**WBF**	1	71	0	3	11	196
	2	65	0	5	4	88
	3	75	1	1	0	169
	4	68	0	0	3	167
**N**_**2**_ **F**	1	70	>10	>10	N/A	71
	2	67	2	>10	N/A	100
	3	69	3	>10	N/A	59
	4	68	3	0	N/A	98
**CO**_**2**_	1	62	N/A	N/A	47	290
	2	60	N/A	N/A	33	286
	3	59	N/A	N/A	51	290
	4	59	N/A	N/A	42	284

### Pig weights, elapsed time from death to post-mortem exam, and post-mortem lesion data

The mean weights of pigs as shown in Supplemental Fig 2A were indicative of a difference in distribution of the weights in each group; however, no statistical between groups were observed. For all methods, the average time lapse between confirmed death and postmortem exam was 84 min, but the time lapse was not consistent between groups (Supplemental Fig 2B and Supplemental Table 2); yet, despite the inconsistencies, there was no significant effect of time lapsed on amount of froth or lung lesions (Supplementary Tables 2 and 3).

### Distribution and amount of foam in the trachea

**Fig 3:**
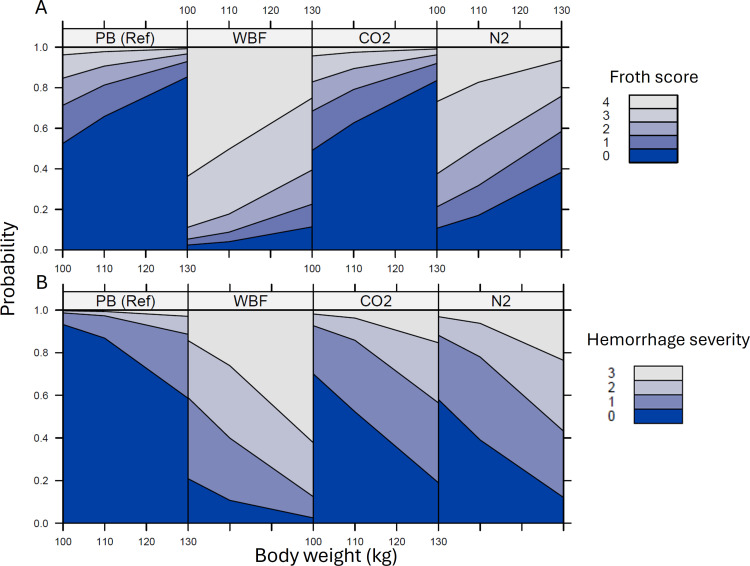
A plot of the effect of method and body weight on the probability of a pig having a given pulmonary edema froth score (A) or alveolar hemorrhage score (B). Froth scores of 0 (royal blue) =  no froth, i.e., dry tracheal lumen; 1 (medium blue) =  froth present but not occluding the lumen, 2 (light blue) = froth present but partially occluding the lumen and tracheal bifurcation still visible, 3 (grey) =  froth present, occluding the lumen, and to a level <  1 cm above the bifurcation, and 4 (light grey) =  froth present, lumen fully occluded, and to a level > 1 cm above the bifurcation. Alveolar hemorrhage scores of 0 (royal blue) =  no lesions, 1 (medium blue) =  mild, 2 (light blue) =  moderate, and 3 (grey) =  severe. Pigs with weights below 100 kg (N =  2) were omitted due to low sample size.

The predicted likelihood of having froth in the trachea was greater for WBF (log-odds =  3.81) and N_2_F (log-odds =  2.24) pigs compared to PB and CO_2_ pigs (Fig 3A). Furthermore, having froth in the trachea was strongly affected by method (*P < * 0.001 per analysis of deviance), whereas body weight tended to have little effect (*P* =  0.09) (Fig 3A). However, not all pigs exposed to foam had froth in the trachea, i.e., a froth score >  0 (Fig 3A). There were two pigs in the WBF group that had unobstructed/ dry tracheas (Foam Score =  0) even though the majority of the WBF group pigs (14/ 25) had total occlusion of the trachea by froth (froth score =  4). Foam in the trachea was notable for all method groups and there was at least one pig in all method groups that had foam occluding the distal trachea and obscuring the tracheal bifurcation (froth score =  3) (Fig 3A).

### Post-mortem lung lesions

The probability of having a higher severity of alveolar hemorrhages increased with body weight; *P* <  0.01, Fig 3B). Of the ten microscopic lung lesion categories, the presence and severity of alveolar hemorrhages were most affected by method with WBF pigs showing a higher level of severe lesions compared to the other methods (*P* <  0.001). There was no difference in alveolar hemorrhage severity between PB, N_2_F or CO_2_ pigs (Fig 3B). There was no effect of the time elapsed between death and examination (*P* =  0.11).

Strong relationships (*P* >  0.5) were identified most frequently for those lesions’ characteristic of occlusion hypoxia, i.e., alveolar hemorrhage, interstitial edema, congested alveolar septa, froth, and emphysema, corroborating the Chi-squared test (Table 4) and the log-odds for alveolar hemorrhages. Therefore, if the lung lesion of alveolar hemorrhage was present and severe, it was most likely due to the treatment causing occlusion hypoxia.

**Table 4:** Chi-square (*χ*^2^) test results for differences in the presence and severity (score) for alveolar hemorrhage depending on the presence of concomitant pneumonia post-mortem.

Lesions as covariates	χ2	*P*-value
Alveolar Edema	16.7266	0.0008
Septal Edema	10.9484	0.012
Lobular Septal Hemorrhage	7.9124	0.0479
Alveolar dilation	7.6512	0.0538
Alveolar Hemorrhage	6.1116	0.1063
Interstitial Edema	5.672	0.1287
Congested alveolar septa	3.1911	0.3631
Froth	4.1527	0.3857
Emphysema	1.5374	0.6737

## Discussion

Both WBF and N_2_F demonstrated success equivocal to CO_2_ in depopulating swine, warranting consideration for their approval for swine depopulation. When formulating emergency depopulation plans, it is important to consider the efficiency and efficacy, as well as availability and ease of use of a particular method. However, other critical factors, including welfare aspects, along with resource availability, protocol safety, and cost, among others, should be considered in the decision-making process.

### Method logistics

In this study, both WBF and N_2_F groups used the same trailer for the depopulation process. The mean differences in total, level of head, and level of shoulder between these two methods were small, and they may be attributable to differences in the equipment used for each method’s foam generation and initiation (i.e., power, hose diameter) or the discrepancies in protocol optimization such as loading animals, and equipment preparation to maximize efficiency. The approach to CO_2_ trailer filling differed in that a sealed chamber is filled using a vaporizing system converting liquid CO_2_ into a gaseous form until the desired CO_2_ level is achieved. Thus, a separate fully enclosed trailer was used for CO_2_ replicates. The total fill time for CO_2_ took approximately five times as long as that of medium-expansion WBF and nearly four times as long as that of high-expansion N_2_F. Overall, fill times for any method will vary depending on the size of the animal chamber relative to the capacity of equipment used to generate and disperse each depopulating agent. An additional factor to consider is operator familiarity and method optimization, as deficiencies in either will undesirably prolong the depopulation process. Therefore, to best expediate the process of both fill times, it will be necessary for an individual to weigh the power of their generating equipment versus appropriate chamber size, as well as implement protocol trainings, to best suit their herd numbers.

### Time to cessation of movement

One key parameter when evaluating the animal welfare of a depopulation method is the rapidity of inducing loss of consciousness in an animal prior to death. A previous study investigating time to loss of consciousness using WBF and electroencephalographic (EEG) data for nursery pigs found that the range for the time to unconsciousness was between 80 and 250 s [31]. Cessation of movement, which has previously been used as a conservative proxy for the onset of unconsciousness [15–18], has been reported to occur well after unconsciousness based on EEG results in pigs [31]. Our reported times to COM were similar in range to previous findings in finisher pigs and cull sows depopulated with WBF [15,17,18], CO_2_ used in dip-lift systems [11] and neonatal pigs during animal chamber conditions [7]. In this aspect, we found no difference in time to COM between WBF and N_2_F but both foam-methods demonstrated a shorter time to COM compared to CO_2_. This outcome is not surprising as the CO_2_ method included a 5-minute CO_2_ displacement protocol, rendering an uneven comparison. However, it was interesting to see that, although no significant difference was observed for the time to COM between WBF and N_2_F, a numerical difference existed, where the mean time to COM was about 40 s shorter for N_2_F. Although not statistically detected in our study, there are suggested advantages of infusing foaming agents used for depopulation with N_2_-gas, due to its quick release and contribution to create anoxic environments which decreases the time to COM [15,24,32–34]. That said, this study shows that the current recommended protocol for large scale CO_2_ depopulation in swine does come with a longer period where pigs display movement compared to both WBF and N_2_F. Finally, the manual recording of the last audible pig movements reflects the calculated COM pattern for each method which was derived from the bio-loggers. This adds reassurance that the used bio-loggers and COM calculations provide a more sensitive and reliable measurement compared to what can be obtained manually.

### Intensity of movement

Looking at EA levels for each of the depopulation methods, pigs in the N_2_F group displayed most of their movement prior to foam initiation, with a peak during foam initiation, followed by a rapid decrease in EA around 30 s post-initiation. This EA pattern was likely linked to the previously mentioned degree of auditory disturbance from starting up the N_2_F generating system prior to the initiation of foam, with the rapid drop in EA caused by the release of N_2_ as bubbles burst around the pigs. Interestingly, although the CO_2_ system also generated enough noise to startle pigs, a similar pattern of EA was not seen until CO_2_ was released into the trailer, with the EA peak occurring at 30 s post-CO_2_ initiation, followed by a slower reduction in EA levels for the next 90 s. It is possible that some of the elevated activity levels represent a physiological surge in muscular excitation prior to or following loss of consciousness, which is often seen in swine euthanized using CO_2_ [8,26]. This muscular excitation may also have affected our calculations for COM for CO_2_ pigs depending on the effect caused on the bio-logger and should therefore be interpreted conservatively. For WBF, increased EA levels started approximately 30 s prior to foam initiation and decreased rapidly within the first minute post-initiation. The increase in EA for WBF coincides with the staff on top of the trailer approaching the open canopy with their aspirated nozzles, which may have elicited behavioral responses from the pigs. Thus, when comparing the EA levels between methods, the absence of visual cues for pigs in the CO_2_ system may be beneficial in reducing initial activity levels prior to CO_2_ release compared to WBF and N_2_F. Moreover, CO_2_ pigs displayed an EA level above the mean threshold 75 s longer compared to the N_2_F pigs and 60 s longer compared to the WBF pigs. In this aspect, one must carefully consider the tradeoff between a shorter but potentially higher intensity of the aversive experience versus a gradually increasing but longer duration of the pigs’ experience.

The data and field observations gained from this study also suggest there are potential methodology improvements to ameliorate pig stress levels in the future, such as investigating equipment modifications to reduce noise levels, which may otherwise exacerbate animal distress. Finally, it is possible that factors associated with physical irritation of the conjunctiva or other mucus membranes, could differ between foams and CO_2_.Therefore, this may have elicited different levels of aversive responses or increased behavioral activities during the depopulation process. These are important considerations for further investigations into minimizing pain and distress inflicted during development of depopulation methods.

### Cardiac activity

Evaluating cardiac activity ensures that a method of depopulation is non-recoverable and complete. In this setting, evaluation of cardiac activity facilitates confirmation of animal death by identifying the presence of a fatal arrhythmia. One pig was determined to be deceased on physical examination while cardiac activity was still present. Our results indicate that WBF and N_2_F predominantly result in ventricular fibrillation (70.4% and 76.9%, respectively) while CO_2_ predominantly resulted in asystole (75%). Other studies in pigs suggest that increased parasympathetic tone may reduce the probability of ventricular fibrillation [35] but also promote the development of asystole [36]. Previously, it has been reported that WBF resulted in a higher frequency of asystole in nursery pigs and cull sows (75% and 55%, respectively) [17]. The frequency of arrhythmias in these instances is believed to be less important, as all arrhythmias were interpreted as terminal. In this study, the lack of a significant difference in time to fatal arrhythmia between the groups suggests that WBF, CO_2_, and N_2_F may all serve as viable depopulation methods.

### Swine behavior

Our results showed that CO_2_ depopulation elicited more vocalizations compared to foam-based methods. Previous studies have stated that mucosal irritation and “air hunger” can occur in mammals and poultry upon CO_2_ inhalation, and this may have contributed to the frequency of vocalizations in the CO_2_ group [2,37]. In addition, the CO_2_ trailer’s fully enclosed design to maximize CO_2_ concentrations may have contributed to additional aversive behaviors, such as vocalization, in comparison to an open canopy design [38,39]. Moreover, the CO_2_ and N_2_F systems may have caused additional pig distress due to novel auditory stimuli prior to agent initiation as both systems had a longer and louder start-up process compared to WBF. This is also reflected in the higher number of observed escape attempts in N_2_F pigs that may have been caused by an elevated state of vigilance compared to WBF pigs. Overall, the present study findings align with other studies that discuss how CO_2_ gas euthanasia can elicit avoidance behaviors, along with negative behavioral responses [2,40].

Water-based foam had fewer instances of resurfacing and escape attempts compared to N_2_F. A slightly slower fill time for N_2_F may have presented additional opportunity for pigs to resurface, as only one resurfacing was observed across all WBF replicates while multiple resurfacings were observed consistently across each N_2_F replicate. Our research group has previously issued recommendations for a minimum fill height for WBF of twice the height of the dorsal apex of the pig’s back, to reduce the possibility of resurfacing [18]. As both the WBF and N_2_F methods rely on rapid foam coverage, the key factor to minimizing chances of resurfacings would be to ensure a high foam fill rate. Since the completion of the study, a commercial AES N_2_F depopulation unit with dual foam generators have been developed, allowing for reduced fill time of the trailer, and increased and unison foam coverage of the pigs. This setup is a promising development to provide less opportunity for pigs resurfacing during the fill, and in extension reducing time to COM, and cessation of cardiac activity.

### Equipment and supply considerations

The availability and accessibility of required resources will be essential for responding to unpredicted emergencies in a timely manner. Ideally, in addition to all other considerations, an emergency depopulation plan should remain suitable per geographical region and be within reasonable cost for the end-user. The PHOS-CHEK WD881 Class A WBF used in our study is a widely commercially available foam concentrate used for forest fires and in the USDA Animal and Plant Health Inspection Service (APHIS) national veterinary stockpile for poultry depopulation. The WBF water pump system is composed of parts readily available in large retail stores and general stores and does not rely on specialized equipment. While the prepared mixture achieves a foam to water expansion ratio of 40 to 50:1, water requirements are approximately 25 L/m^3^ of container space used, requiring an ample and reliable water source during operation. The N_2_F requires a foam-to-water expansion ratio of 300 to 350:1 significantly reducing the water requirements. Carbon dioxide gas would be a more feasible option in regions where water is generally sparse, during droughts, or where it is not practical or possible to obtain large quantities of water. However, as evidenced in the United States and Europe in 2022, the CO_2_ supply is susceptible to seasonal shortages, risking regional inaccessibility to end-users [41].

### Post-mortem lesions

When the analyses of the distribution and amount of froth in the tracheas of the pigs in each group are taken together, two trends are clearly shown. First, the PB method and the CO_2_ are very unlikely to have pigs with froth occluding the tracheas as inherent with the dry/non-foam methods and they are comparable to each other. Second, pigs of higher body weights are more likely to have clear tracheas which suggests that death in heavier pigs is due to anoxic-induced brain cellular death rather than occlusion hypoxia. Lung lesions of alveolar hemorrhage and presence of froth occluding the trachea were findings most often associated with pigs in the WBF group which suggest that in this group, occlusion hypoxia was the cause of death. Lung lesions such as alveolar hemorrhage, emphysema, alveolar edema, alveolar septal congestion, interstitial edema, septal edema, alveolar dilation, and lobular septal hemorrhages were scored and recorded since these were our lesions of interest and can be present to different degrees and severity depending on cause of death [29,42]. However, some of these lesions can also be associated with porcine pneumonia. There were pigs in all method groups and in the PB group that had froth in the trachea to varying degrees and severity. The presence of froth post-mortem is an indicator of pulmonary edema which is attributable to death due to barbiturates and opiate overdoses, drowning, congestive heart failure, and trauma per human and veterinary forensic pathologists [30,43,44]. When all method groups’ lung lesions scores were compared to the PB group, only CO_2_ group pigs were considered comparable, but pigs in the N_2_F group had many similarities to the CO_2_ group. These findings suggest that death occurring by CO_2_, N_2_F and PB are equivalent when only considering mechanism of death as the outcome. However, when all measures are considered together, all four methods, including WBF, when performed proficiently, are irreversible and repeatable.

### Study limitations

We note that the study pigs were assigned to their specific depopulation method based on convenience and that replicates for each methodology were performed sequentially, rather than through randomization. Due to the inherent nature of this being a field study, there were differences between containers for each depopulation method examined, which resulted in the inability to compare all animal-level assessments across methods. Additionally, we want to highlight that the assessment of cessation of movement as a proxy for loss of consciousness is conservative. The N_2_F group examined post-mortem was smaller compared to WBF and CO_2_ due to unplanned logistical constraints, and results must be interpreted accordingly. It is also important to note that this study was conducted during one point-in-time, and a variety of environmental conditions or seasonality were therefore not considered. Finally, the movement of the pigs from the containers to the necropsy areas may have created post-mortem artifacts such as movement of foam out of or into the respiratory tract, thereby further adding to the subjectivity of the foam scores.

## Conclusions

The selection of a depopulation method is multifaceted and will vary by end-user and their swine herd. However, the performance of WBF and N_2_F was well within the parameters of the currently recommended CO_2_ option for swine depopulation and provided shorter times to cessation of movement and similar times to fatal arrhythmias. Each method provided a 100% mortality rate with no signs of regained consciousness during or after the assigned dwell-time period. Based on the findings of this study, the use of WBF or N_2_F would be suitable option for large-scale depopulation of swine.

Overall, this study’s findings will help inform future decisions on developing emergency contingency plans. Additional research on public and swine industry stakeholders’ perceptions on these studied methods, along with potential effects that foam-based methods have on pathogens and environment long-term, are next steps that should be investigated.

## Supporting information

S1 Table 1 Spearman’s *ρ* statistic for lung lesions scores as ordinal covariates. Lesions considered characteristic of mechanical asphyxiation are indicated by asterisks (*). Lesions with strong correlation scores ( ≥ 0.5 *ρ* ≤  1.00) are in bold highlighted.
(DOCX)

S2 Table 2 Time lapsed (min) between confirmed death and post-mortem exam. N2 =  Nitrogen gas-filled foam group; CO2 =  Carbon dioxide gas group, WBF =  Water-based foam group; and PB =  pentobarbital (referent) group. ALL =  all groups combined.
(DOCX)

S3 Table 3 The change (±SE) in log-odds for water-based foam (WBF), nitrogen foam (N_2_F), and carbon dioxide (CO_2_) compared to the referent pentobarbital (PB) group for observations of froth in the trachea by method, body weight and time elapsed between time of confirmed death and post-mortem examination. (DOCX)

S4 Fig 1 Number of pigs with pulmonary edema froth scores of 0, 1, 2, 3, or 4 by method (PB =  pentobarbital, WBF =  Water-based foam, CO_2_ =  Carbon dioxide gas, N_2_F =  nitrogen foam). Froth scores of 0 =  no froth (dry tracheal lumen), 1 =  froth present but not occluding the lumen, 2 =  froth present but partially occluding the lumen and tracheal bifurcation still visible, 3 =  froth present, occluding the lumen, and to a level <  1 cm above the bifurcation, and 4 =  froth present, lumen fully occluded, and to a level >  1 cm above the bifurcation.
(TIF)

S5 Fig 2 Exploratory plots of antemortem body weights (A) in kilograms (±SD) and time lapsed (min) between time of confirmed death and post-mortem examination (B) by method (N2F =  Nitrogen foam; CO2 =  Carbon dioxide gas; WBF =  Water-based foam; and PB =  pentobarbital (referent). (TIF)
