# Supplementary material for: Assessment of three large-scale depopulation methods for swine
Source: PLoS One. 2025 Mar 25;20(3):e0320217. doi: 10.1371/journal.pone.0320217 (PMC11936211; doi:10.1371/journal.pone.0320217)
Supplement: S1 Table 1 — Lesions considered characteristic of mechanical asphyxiation are indicated by asterisks (*). Lesions with strong correlation scores ( ≥ 0.5 ρ ≤ 1.00) are in bold highlighted. (DOCX) [file pone.0320217.s001.docx]

**S1 Table 1.** Spearman’s *ρ* statistic for lung lesions scores as ordinal covariates. Lesions considered characteristic of mechanical asphyxiation are indicated by asterisks (*). Lesions with strong correlation scores (≥ 0.5 *ρ* ≤ 1.00) are in bold highlighted.

|  | **Foam*** | **Emphysema*** | **Alveolar hemorrhage*** | **Congested alveolar septa*** | **Interstitial edema*** | **Septal Edema** | **Lobular Septal Hemorrhage** | **Alveolar dilation** | **Alveolar edema** |
| --- | --- | --- | --- | --- | --- | --- | --- | --- | --- |
| *Foam** | 1.000 | 0.258 | 0.422 | 0.090 | 0.432 | 0.280 | 0.252 | 0.329 | 0.148 |
| *Emphysema** | 0.258 | 1.000 | 0.187 | -0.029 | 0.392 | 0.101 | 0.329 | 0.284 | 0.127 |
| *Alveolar hemorrhage** | 0.422 | 0.187 | 1.000 | **0.546** | **0.835** | **0.565** | **0.717** | 0.424 | **0.522** |
| *Congested alveolar septa** | 0.090 | -0.029 | **0.546** | 1.000 | 0.441 | 0.357 | 0.489 | 0.313 | **0.612** |
| *Interstitial edema** | 0.432 | 0.392 | **0.835** | **0.441** | 1.000 | **0.550** | **0.840** | 0.447 | 0.422 |
| *Septal Edema* | 0.280 | 0.101 | **0.565** | 0.357 | **0.550** | 1.000 | **0.611** | **0.638** | 0.380 |
| *Lobular Septal Hemorrhage* | 0.252 | 0.329 | **0.717** | 0.489 | **0.840** | **0.611** | 1.000 | 0.436 | 0.467 |
| *Alveolar dilation* | 0.329 | 0.284 | 0.424 | 0.313 | 0.447 | **0.638** | 0.436 | 1.000 | 0.350 |
| *Alveolar edema* | 0.148 | 0.127 | **0.522** | **0.612** | 0.422 | 0.380 | 0.467 | 0.350 | 1.000 |
