# Supplementary material for: Assessment of three large-scale depopulation methods for swine
Source: PLoS One. 2025 Mar 25;20(3):e0320217. doi: 10.1371/journal.pone.0320217 (PMC11936211; doi:10.1371/journal.pone.0320217)
Supplement: S2 Table 2 — N2 = Nitrogen gas-filled foam group; CO2 = Carbon dioxide gas group, WBF = Water-based foam group; and PB = pentobarbital (referent) group. ALL = all groups combined. (DOCX) [file pone.0320217.s002.docx]

**S2 Table 2**. Time lapsed (minutes) between confirmed death and post-mortem exam. N_2_ = Nitrogen gas-filled foam group; CO_2_ = Carbon dioxide gas group, WBF = Water-based foam group; and PB = pentobarbital (referent) group. ALL = all groups combined.

| **Method Group** | **Average** | **Minimum** | **Maximum** | **Standard Deviation** |
| --- | --- | --- | --- | --- |
| N2 | 42 | 2 | 95 | 34 |
| CO2 | 96 | 2 | 190 | 55 |
| WBF | 74 | 14 | 153 | 37 |
| PB | 122 | 19 | 169 | 42 |
| **ALL** | **84** | **2** | **190** | **49** |
