# Supplementary material for: Assessment of three large-scale depopulation methods for swine
Source: PLoS One. 2025 Mar 25;20(3):e0320217. doi: 10.1371/journal.pone.0320217 (PMC11936211; doi:10.1371/journal.pone.0320217)
Supplement: S3 Table 3 — (DOCX) [file pone.0320217.s003.docx]

**S3 Table 3.** The change (±SE) in log-odds for water-based foam (WBF), nitrogen foam (N_2_F), and carbon dioxide (CO_2_) compared to the referent pentobarbital (PB) group for observations of froth in the trachea by method, body weight and time elapsed between time of confirmed death and post-mortem examination.

|  | **Log-odds** | ±**SE** | **t-value** |
| --- | --- | --- | --- |
| WBF | 3.81 | 0.94 | 4.07 |
| CO_2_ | 0.14 | 0.82 | 0.17 |
| N_2_ | 2.24 | 1.03 | 2.17 |
| Body Weight | -0.06 | 0.04 | -1.57 |
| Time Elapsed | 0.005 | 0.006 | 0.80 |
