# Supplementary figures and images for: Assessment of three large-scale depopulation methods for swine
Source: PLoS One. 2025 Mar 25;20(3):e0320217. doi: 10.1371/journal.pone.0320217 (PMC11936211; doi:10.1371/journal.pone.0320217)

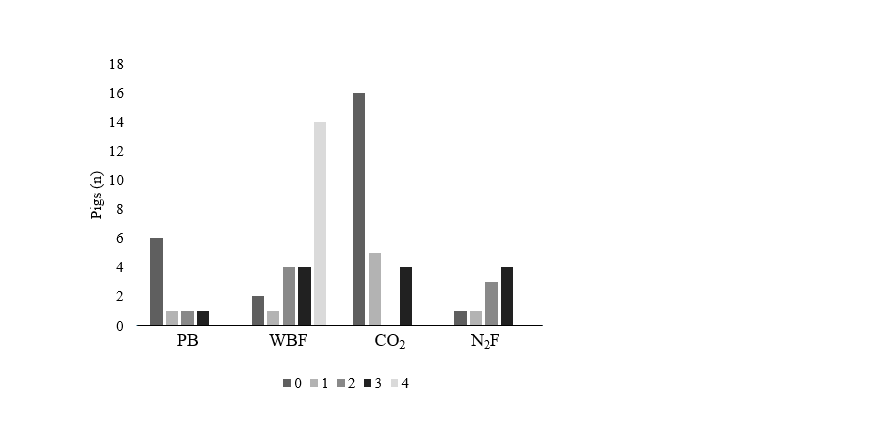

Supplement: S4 Fig 1 — Froth scores of 0 = no froth (dry tracheal lumen), 1 = froth present but not occluding the lumen, 2 = froth present but partially occluding the lumen and tracheal bifurcation still visible, 3 = froth present, occluding the lumen, and to a level < 1 cm above the bifurcation, and 4 = froth present, lumen fully occluded, and to a level > 1 cm above the bifurcation. (TIF) [file pone.0320217.s004.tif]

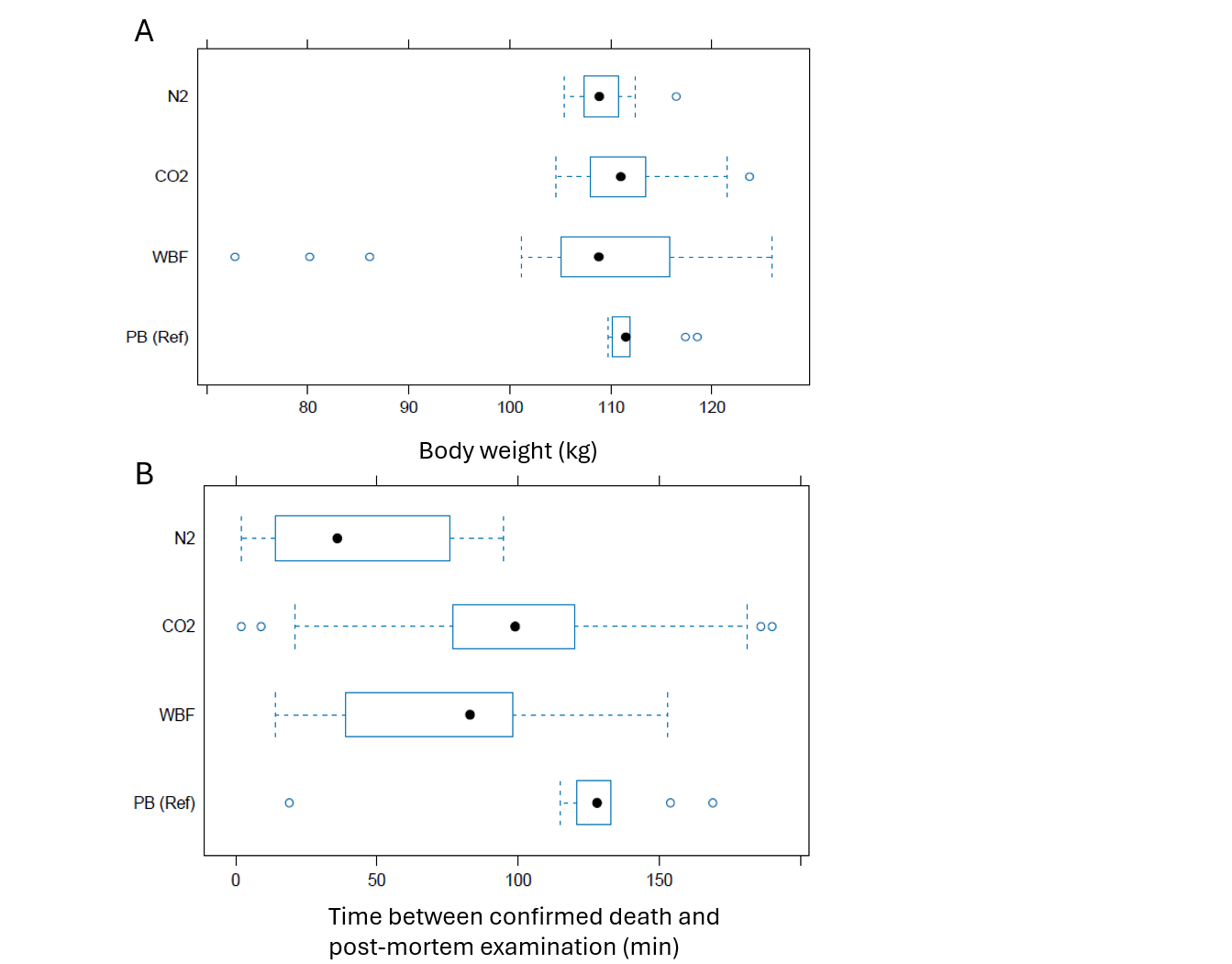

Supplement: S5 Fig 2 — (TIF) [file pone.0320217.s005.tif]
